# Exploring the Complex Pathways between the Fear of COVID-19 and Preventive Health Behavior among Nigerians: Mediation and Moderation Analyses

**DOI:** 10.4269/ajtmh.20-0994

**Published:** 2021-07-08

**Authors:** Olusola Ayandele, Cristian A. Ramos-Vera, Steven K. Iorfa, Catherine O. Chovwen, Peter O. Olapegba

**Affiliations:** 1 Department of General Studies, The Polytechnic, Ibadan, Nigeria;; 2 Research Department, Universidad Cesar Vallejo, Lima, Perú;; 3 Department of Psychology, University of Nigeria, Nsukka, Nigeria;; 4 Department of Psychology, University of Ibadan, Ibadan, Nigeria

## Abstract

Since COVID-19 currently has no proven cure but high morbidity and mortality, many people are living in fear of the virus along with other mental health challenges induced by the lockdowns and social distancing. Hence, this study aims to provide evidence on the co-occurrence and inter-relations between the fear of COVID-19, post-traumatic stress symptoms, and psychological distress in adherence to preventive health behavior among Nigerians. It also seeks to determine whether this process differs for men and women. The sample comprised 1,172 consenting young adults (mean age = 22.9 ± 6.6 years, 54.5% females) selected using a snowball sampling technique. Structural equation modeling (SEM) was used to test the mediation model of post-traumatic stress symptoms and psychological distress as parallel and serial mediators of the relationship between the fear of COVID-19 and preventive health behavior. The indirect effect of the fear of COVID-19 on preventive health behavior across gender was tested using moderation analysis. Results showed that post-traumatic stress symptoms and psychological distress serially and fully, in causal order, mediated the association between the fear of COVID-19 and preventive health behavior, and gender moderated the mediation effects. The research provides evidence that the fear of COVID-19 could trigger preventive health behavior through post-traumatic stress symptoms but reduces it through psychological distress, whereas the fear of COVID-19 has a slightly more positive impact on preventive health behavior among men.

## INTRODUCTION

The outbreak of a novel coronavirus, the severe acute respiratory syndrome coronavirus-2 (SARS-CoV-2), in Wuhan, China, in late December 2019 quickly became the COVID-19 pandemic, which has led to significant physiological and mental health challenges in all parts of the world. The COVID-19 pandemic confirmed that an infectious disease can travel from one continent to another in a matter of hours. As of August 9, 2020, there were 19,718,030 cases of COVID-19 resulting in 728,013 deaths worldwide, whereas Nigeria had 46,867 confirmed cases and 950 deaths.^1^ Because of the high rate of SARS-CoV-2 contagiousness and the morbidity and mortality of COVID-19, both the infected and uninfected healthy people are terror-stricken and living under the fear of infectivity of the virus.^2^ Similarly, conspiracy theories and misinformation about the sources, transmission, prevention, and cure of COVID-19 as well as the psychosocial and economic effects of social distancing and lockdown exposed many in Nigeria (and elsewhere) to physical and mental health challenges.^2^^–^^5^

As efforts at establishing effective treatments and developing a vaccine for the disease are ongoing, preventive health behavior is currently the major means of curbing the spread of the novel coronavirus.^6^ Preventive health behavior refers to the various infection prevention and control practices that can effectively mitigate the spread of the coronavirus. The World Health Organization advises people to practice hygiene etiquette, to regularly wash or sanitize their hands, to wear face masks, to stay indoors, to avoid physical contact with other people, to self-isolate if having symptoms of COVID-19 and visit a health center, and to regularly disinfect possibly contaminated objects or surfaces.^6^^,^^7^ Many countries implemented lockdown and social distancing rules, conducted extensive testing and contract tracing, and quarantined symptomatic and asymptomatic persons while those having close contact with infected persons were isolated. A combination of personal hygiene, social distancing, disinfecting, and quarantine may prevent airborne transmission of aerosols carrying COVID-19 and avert contact transmission of coronavirus droplets on surfaces.^8^ Earlier studies had identified greater knowledge about COVID-19 to be linked to higher compliance with social distancing and COVID-19 hygiene etiquette.^9^^,^^10^ However, social distancing, lockdown measures, and the relatively deadly nature of the COVID-19 may pose risks to the mental health of people.^11^

Taylor suggested that an outbreak of an infectious disease could lead people to cultivate anxiety-related distress behaviors such as fear of infection, fear of isolation, fear of strangers, and post-traumatic stress.^12^ The fear of COVID-19 may be seen from the wide range of fear-related behaviors such as belief in conspiracy theories,^5^ and precipitating psychological distress,^11^ substance abuse,^13^ and different mental illness.^14^ However, fear is also likely to encourage a reduction in risky behaviors.^15^^,^^16^ Research showed that individuals with higher fear of COVID-19 across Europe and America were more likely to engage in preventive health protocols such as social distancing and hand washing because of the fear of infection.^17^^,^^18^

Research has also suggested that infectious diseases are likely to contribute to psychological distress and consequently poor mental health.^14^^,^^16^ Psychological distress refers to the general emotional disturbance expressed through negative mood, anxiety, and stress.^19^ A survey conducted among Ebola virus disease patients and their relatives in Nigeria revealed high levels of psychological distress among the respondents, virtually all the patients reported an inability to concentrate and losing much sleep over worry.^20^ Since the current outbreak, there have been reports of heightened mental health problems and psychological distress among the general public affected by the COVID-19 outbreak in various countries.^4^^,^^11^^,^^13^

The relationship between the fear of COVID-19 and preventive health behavior can be further corroborated by the presence of symptoms of post-traumatic stress. Experience of traumatic events such as rape, accidents, mass conflicts, epidemics, or natural disasters is most frequently associated with symptoms of post-traumatic stress disorder (PTSD).^12^^,^^21^ The COVID-19 pandemic can be perceived as a traumatic event as it meets some key diagnostic criterion of PTSD in the fifth edition of the *Diagnostic Statistical Manual of Mental Disorder* (*DSM 5*); it has persisted over time (since the beginning of the year 2020), threatened people’s lives, and interfered with their social and occupational functions (social distancing and lockdown).^22^ Studies have revealed the existence of symptoms of post-traumatic stress among COVID-19 patients and the general public.^23^

### The present study.

Based on the novel nature of the coronavirus disease and its negative impacts on Nigerians’ mental health status, as well as the psychological effects of the fear of infectivity of the virus, engaging in preventive behavior and abiding with social distancing rules, more evidence-based studies on how the relationships between demographics, mental health status, and the fear of COVID-19 relates to preventive health behavior are needed. Besides, Nigeria, being a non-WEIRD society, is not well represented in the literature on the relationship between mental health during the COVID-19 pandemic.^2^^,^^3^^,^^7^^,^^20^^,^^24^ To address this gap, the current study aimed to specifically test whether the relationship between the fear of COVID-19 and preventive health behavior is mediated by post-traumatic stress symptoms and psychological distress, and moderated by gender, during the COVID-19 outbreak in a Nigerian sample. Four hypotheses were formulated:

H1: The fear of COVID-19 will be positively associated with preventive health behavior.

H2: Post-traumatic stress symptoms and psychological distress will in parallel mediate the relationship between the fear of COVID-19 and preventive health behavior.

H3: The relationship between the fear of COVID-19 and preventive health behavior will be serially mediated by post-traumatic stress symptoms and psychological distress.

H4: Gender will moderate the effect of the fear of COVID-19 on preventive health behavior among Nigerians.

**Figure 1.**
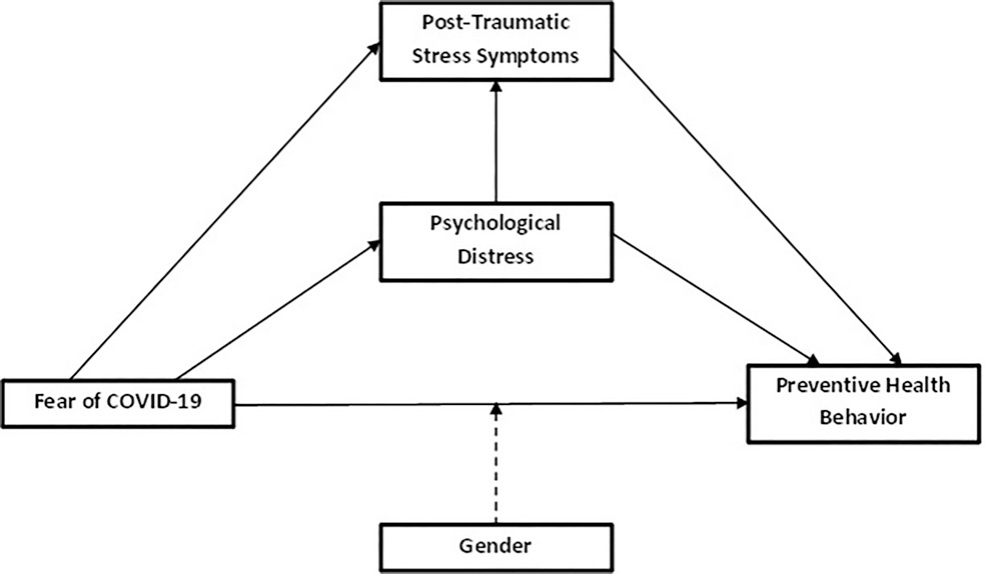
Conceptual model of the expected moderated mediation showing the indirect, direct, and total effects (continuous lines) and the moderating effects (dotted lines).

A summary of the hypotheses and conceptual model of the expected moderated mediation is described in Figure 1.

## MATERIALS AND METHODS

### Participants and procedure.

The restrictive governmental measures of social distancing and movement necessitated the use of online survey methodology. Participants were recruited using a snowball sampling technique via WhatsApp, Telegram, Facebook, and Google groups of students in The Polytechnic Ibadan, Nigeria, and they were encouraged to invite their family and friends to participate. Factors considered for the selection included proximity to the researchers, access to the Internet, providing informed consent, and so on. Google form was used to host the survey questions and the link shared on social media by the researchers. The study was conducted in compliance with the Declaration of Helsinki. Informed consent was obtained from all participants included in the study. Participation was voluntary, anonymous, and confidential. Participants were assured that they are at liberty to withdraw from the study at any time they so wish. The study did not collect any personal data through which an individual could be identified. Upon completion of the survey, participants were directed to a counseling website to help with likely stress caused by talking about this issue.

### Measurement instruments.

Participants completed a questionnaire that included the aims of the study and information on confidentiality and voluntary participation, and 43 items that covered five areas: sociodemographic factors (gender, age, relationship status, educational qualification, socioeconomic status, and religion), preventive health behavior, the fear of COVID-19, post-traumatic stress symptoms, and psychological distress.

The Infectious Diseases Preventive Health Behavior Scale (ID-PHBS) was used to assess the levels of compliance with recognized preventive protocol against infectious diseases such as COVID-19.^25^ A 7-point response scale (1 = *strongly disagree*, 7 = *strongly agree*) was used to measure items such as “I frequently sanitize/wash my hands with soap and water.” A composite preventive health behavior score is calculated by adding up each of the 12 item’s scores (ranging from 12 to 84). Higher scores indicate the greater practice of preventive health behavior. The authors reported a reliability coefficient of 0.82. Its goodness of fit indices were found to be acceptable (χ^2^/df = 3.64, GFI = 0.949, CFI = 0.975, TLI = 0.983, RMSEA = 0.061, and SRMR = 0.068) and the Cronbach’s alpha observed in the present study was 0.85.

In the third part, the fear of COVID-19 scale (FCV-19S), a seven-item one-dimensional scale, was used to measure the fear of COVID-19 among the general population.^2^ The seven items (e.g., “When I watch news and stories about coronavirus 2019 on social media, I become nervous or anxious”) are rated on a 5-point scale from 1 (strongly disagree) to 5 (strongly agree) with scores ranging from 7 to 35. Higher scores mean greater fear of COVID-19. Ahorsu et al.^2^ reported internal consistency of 0.82. Cronbach’s alpha observed in the present study was 0.84.

The fourth section of the questionnaire contains the General Health Questionnaire-12 (GHQ-12), a screening instrument for “psychological distress” among the general public.^19^ GHQ-12 contains 12 items, six of the items are positively worded and the other six are negatively worded. Only the six negatively worded items were used for analysis. Scoring ranges from 0 to 18 using a Likert scale (0-1-2-3). Higher scores on the instrument imply a greater probability of psychological distress. A Cronbach’s alpha of 0.75 and 0.79 has previously been established in a Nigerian population.^26^ A Cronbach’s alpha of 0.73 was reported in this study.

The last section of the questionnaire included the shortened Impact of Event Scale-6 (IES-6) used to measure post-traumatic stress symptoms.^27^ The IES-6 includes a total of six items (scored from 0 to 24 on a Likert scale ranging from 0 (not at all) to 4 (extremely). It is widely used for screening at-risk patients with PTSD and not to diagnose PTSD in clinical settings. Higher scores are interpreted to indicate a high level of symptoms of PTSD. The shortened IES-6 scale has been used in Nigeria and it demonstrated acceptable internal reliability (Cronbach’s alpha = 0.78).^21^ The internal consistency score observed in this study was 0.70.

### Statistical analysis.

Data were analyzed using the JASP 12.2 (JASP Team, 2020) software package. For the preliminary analysis, descriptive statistics of the demographic data were computed and normal distribution assumptions were verified. The absence of multicollinearity between studied variables, checked by conducting a bivariate correlational analysis, showed a moderately positive correlation coefficient (*r* = 0.76). Arguably, with the correlation coefficient below 0.80, we could proceed with the analysis assuming relative independence among the dependent variables. Similarly, the obtained collinearity statistics (variance inflation factor [VIF] > 10 and tolerance < 0.1) do not reach critical values.^28^ Seeing that the data did not deviate significantly from a normal distribution, correlation analysis using structural equation modeling (SEM) was performed (see Table 2). We tested the structural model using the unweighted least squares.^29^ Several goodness-of-fit indices were used as criteria for the selection of the above model. We used χ^2^/df < 5, GFI, CFI, NFI, TLI > 0.90, SRMR, and RMSEA < 0.08 as the model fit index evaluation standards.^30^ We performed bootstrapping with 5,000 samples for the analysis of moderate mediation considered the conditioning parameter from the mean and standard deviation (–1SD, +1SD) through the statistical package jamovi Advanced Mediation Models (jAMM).^31^ The data used for this study are available at https://osf.io/3bpxf.

**Table 2:** Descriptive statistics, scale reliability, and correlations of the study variables

Variable	1	2	3	4	Mean	SD	SK	KU	α
1. PHB	—				64.55	13.80	−0.79	0.20	0.81
2. FCV-19	0.51**	—			22.74	7.42	−0.22	−0.81	0.84
3. Trauma	0.56**	0.76**	—		14.22	5.18	−0.24	−0.32	0.70
4. Distress	0.11*	0.38**	0.44*	—	7.67	4.12	0.19	−0.42	0.70

PHB = preventive health behavior; FCV-19 = the fear of COVID-19; Trauma = post-traumatic stress symptoms; Distress = psychological distress; SD = standard deviation; SK = skewness; KU = kurtosis; α = Cronbach's alphas.

* *P* < 0.05.

** *P *< 0.01.

## RESULTS

### Demographic characteristics of participants.

Between June 21 and 27, 1,172 participants from all six geopolitical zones (36 states and the Federal Capital Territory) in Nigeria responded to the survey. The majority of participants were women (54.5%), aged between 15 and 21 years (47.2%) (mean = 22.9, SD = 6.6). There were more unmarried persons (51.2%), Christians (61.7%), from a poor financial background (54.4%), and not so educated (61.3%; < college degree) in the sample (Table 1).

**Table 1:** Distribution of sociodemographic data of participants

	Frequency	Percent
Gender		
Female	639	54.52
Male	533	45.48
Age (mean = 22.9, SD = 6.6)		
Younger than 21 years	553	47.18
Older than 21 years	619	52.82
Relationship status		
Single	600	51.20
Dating	572	48.80
Religion		
Christianity	723	61.69
Islam	377	32.17
Others	72	6.14
Socioeconomic status		
Lower	638	54.44
Middle	367	31.31
Upper	167	14.25
Educational qualification		
High school certificate	719	61.35
University degree	453	38.65
Geopolitical zone of origin		
North Central	291	24.83
North East	37	3.16
North West	141	12.03
South East	114	9.73
South-South	123	10.49
South West	466	39.76

SD = standard deviation.

### Preliminary analysis.

The descriptive statistics, reliability, and correlations of our variables are presented in Table 2. The correlation values were calculated using a SEM method. As expected, preventive health behavior was positively associated with the fear of COVID-19 (*r* = 0.51, *P* < 0.01), and post-traumatic stress symptoms (*r* = 0.56, *P* < 0.01), but negatively with psychological distress (*r* = −0.11, *P* < 0.05). Furthermore, the fear of COVID-19 was positively associated with post-traumatic stress symptoms (*r* = 0.76, *P* < 0.01) and psychological distress (*r* = 0.38, *P* < 0.01). Psychological distress was positively associated with post-traumatic stress symptoms (*r* = 0.44, *P* < 0.01). Also, results indicated that skewness ranged from −0.79 to 0.52 and kurtosis ranged from −0.81 to 0.21 and was within the criteria of normality. All reliability coefficients were found to range from 0.70 to 0.84 and therefore acceptable.

### Findings of the research model.

In the SEM analysis, the goodness of fit indices of the study mediation model were found to be significant (χ^2^ (1,828, *N* = 1172) = 418; *P* < 0.001; χ^2^/df = 4.37; GFI = 0.95; CFI = 0. 96; NFI = 0.96; TLI = 0.96; SRMR = 0.069; RMSEA = 0.080.

### Association of the fear of COVID-19 with preventive health behavior.

**Figure 2.**
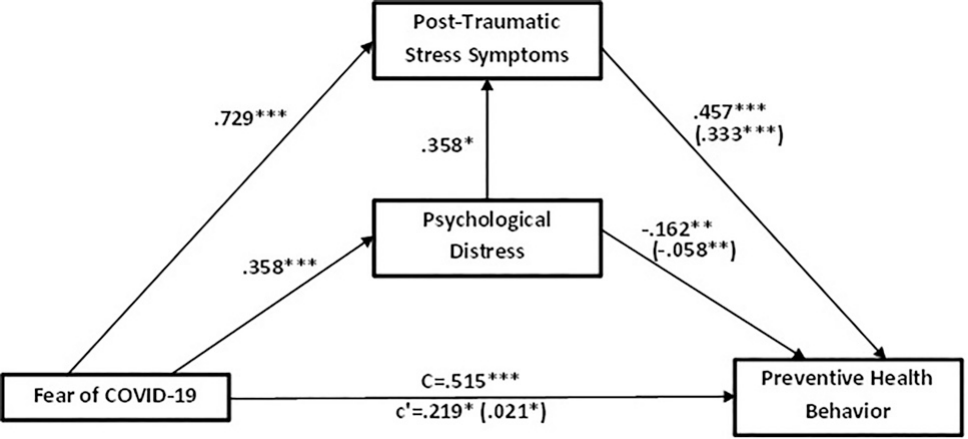
Mediation model (*N* = 1172) of the effects of fear of COVID-19 on preventive health behavior through post-traumatic stress and psychological distress. c’: total effect; C: direct effect and the indirect effects are enclosed (in brackets).

The bootstrapping analysis revealed a positive direct effect of the fear of COVID-19 on preventive health behavior (total effect; β = 0.515, *P* < 0.001). When the two mediating variables were included in the analysis, this coefficient was reduced but was still statistically significant (direct effect, β = 0.219, *P* < 0.05) (Figure 2). This supports the first hypothesis that the fear of COVID-19 will be positively associated with preventive health behavior.

### Parallel mediation model of post-traumatic stress symptoms and psychological distress in the relationship between the fear of COVID-19 and preventive health behavior.

The fear of COVID-19 was a positive predictor of post-traumatic stress symptoms (β = 0.729, *P* < 0.001) and the consequent mediation by post-traumatic stress symptoms had a positive predictive effect on preventive health behavior (β = 0.457, *P* < 0.001) and a significant indirect effect on preventive health behavior (β = 0.333 *P* < 0.001). Simultaneously, the fear of COVID-19 also predicted psychological distress (β = 0.358, *P* < 0.001) and the mediation by psychological distress negatively predicted preventive health behavior (β = −0.162, *P* < 0.01) with the indirect effect of the fear of COVID-19 on preventive health behavior through psychological distress also significant (β = −0.058, *P* < 0.01) (Figure 2). The second hypothesis that post-traumatic stress symptoms and psychological distress will in parallel mediate the relationship between the fear of COVID-19 and preventive health behavior is also supported.

### Serial mediation of post-traumatic stress symptoms and psychological distress between the fear of COVID-19 and preventive health behavior.

Serial mediation hypothesizes a causal chain linking of the mediators (post-traumatic stress symptoms and psychological distress), with a specified direction flow. Added effect of psychological distress on post-traumatic stress symptoms, the relationship was significant with a significant standardized effect (β = 0.128, *P* < 0.01). For example, the fear of COVID-19 could increase post-traumatic stress symptoms, which could in turn increase psychological distress and thus decrease preventive health behavior (i.e., the fear of COVID-19 > post-traumatic stress symptoms > psychological distress > preventive health behavior). The relationship has a significant standardized effect (β = 0.021, *P* < 0.05) (see Table 3). Therefore, hypothesis 3 was also supported.

**Table 3:** Bootstrapping analysis results of the model

Pathway	Coefficient	CL	*P*
Direct effect			
FCV-19 ⇒ Trauma	0.729	0.468–0.735	< 0.001
Traumas ⇒ PHB	0.457	0.493–1.269	< 0.001
FCV-19 ⇒ Distress	0.358	0.209–0.408	< 0.001
Distress ⇒ PHB	−0.162	−0.492 to −0.107	0.002
Distress ⇒ Traumatic	0.128	0.027–0.218	0.012
FCV-19 ⇒ PHB	0.219	0.070–0.618	0.014
FCV-19 ⇒ PHB (Total)	0.515	0.629–1.011	< 0.001
Indirect effect (parallel mediation)			
FCV-19 ⇒ Trauma ⇒ PHB	0.333	0.285–0.776	< 0.001
FCV-19 ⇒ Distress ⇒ PHB	−0.058	−0.157 to −0.028	0.005
Indirect effect (serial mediation)			
FCV-19⇒ Distress ⇒ Trauma ⇒ PHB	0.021	0.002–0.065	0.038
Moderation effect (Gender interactions)			
FCV-19: Gender ⇒ PHB	0.019	0.486–0.070	0.034
Conditional effects of different gender			
Female: FCV-19 ⇒ PHB	0.237	0.239–0.589	< 0.001
Male: FCV-19 ⇒ PHB	0.376	0.474–0.842	< 0.001

CL = confidence limit; *P* = significance; PHB = preventive health behavior; FCV-19 = the fear of COVID-19; Trauma = post-traumatic stress symptoms; Distress = psychological distress. The table shows standardized indirect effects with bootstrapped SEs; ∫Bias-corrected 95% CIs and moderation effects of gender.

### Moderation effect of gender on the fear of COVID-19 on preventive health behavior among Nigerians.

**Figure 3.**
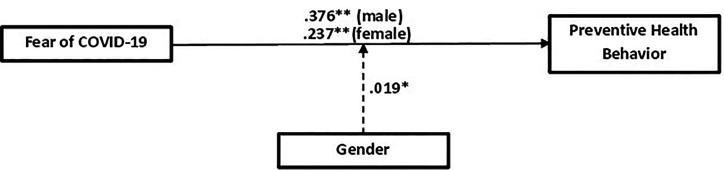
Model of the indirect effects of fear of COVID-19 on preventive health behavior supposedly moderated by gender.

This is to assess if the indirect effect of the fear of COVID-19 on preventive health behavior is moderated by gender. Outcomes in Figure 3 and Table 3 show that gender significantly moderated the effect of the fear of COVID-19 on preventive health behavior (β = 0.019, *P* < 0.05). Also, the fear of COVID-19 recorded a slightly higher positive effect on preventive health behavior for males (β = 0.376; *P* < 0.01) than females (β = 0.237; *P* < 0.01). The stated hypothesis that gender will moderate the effect of the fear of COVID-19 on preventive health behavior among Nigerians is therefore supported with a statistically significant difference between males and females.

## DISCUSSION

Although a plethora of research has explored the patterns of association between preventive health behaviors, the fear of COVID-19, and the mental health status since the start of the pandemic as well as the complex pathways through which they influence each other.^9^^,^^17^^,^^18^ The present study comes as one of the first to explore these relationships in a mediation and moderation approach. Specifically, this study explored the extent to which gender moderates the mediating effect of post-traumatic stress symptoms and psychological distress on the relationship between the fear of COVID-19 and preventive health behavior.

The first hypothesis that the fear of COVID-19 will be positively associated with preventive health behavior is supported. The findings indicated that high levels of the fear of COVID-19 were more likely to trigger engagement in frequent preventive health behavior. The result is consistent with previous studies that identified preventive health behavior as being enhanced by the fear of COVID-19.^9^^,^^15^^,^^17^^,^^18^ In a similar study among Nigerians, a direct effect of risk perception on precautionary health behaviors was reported.^9^ Previous studies have shown that fear may be instrumental in triggering specific problem-solving or problem-avoiding behaviors which in turn may avert the feared incident or situation from happening.^32^ It is worthy of note that while fear increases people’s alertness to the severity of risks, excessive fear could hinder engagement in preventive behaviors.^33^ It is, therefore, necessary not to assume that fear of the pandemic will lead individuals into preventive health behavior but rather engage rapidly in campaigns that promote adequate knowledge and attitude toward preventive health behavior.

The findings from this study further expand upon previous ones by exploring the effects of post-traumatic stress symptoms and psychological distress. As hypothesized (H2), the relationship between the fear of COVID-19 and preventive health behavior was mediated simultaneously through post-traumatic stress symptoms and psychological distress. This means that post-traumatic stress symptoms and psychological distress serve as pathways through which the fear of COVID-19 may be translated into preventive health behaviors, such that higher levels of post-traumatic stress symptoms are likely to lead to higher levels of preventive health behavior; whereas higher levels of psychological distress are more likely to lead to lower levels of preventive health behavior in persons with a fear of COVID-19. Although the mediation of post-traumatic stress symptoms in the relationship between the fear of COVID-19 and preventive health behavior encouraged preventive health behavior, the mediation of psychological distress potentially reduced preventive health behavior. This means that in the presence of post-traumatic stress symptoms, the fear of COVID-19 is more likely to translate into higher levels of preventive health behavior while for individuals experiencing higher levels of psychological distress, the fear of COVID-19 is likely to translate into lower levels of preventive health behavior. The double-edged effect of fear in the presence of post-traumatic stress symptoms and psychological distress demonstrates the need to pay attention to early intervention and prevention of psychological disorders among the general public, as the world tries to curb the pandemic.^23^ It is also important to be knowledgeable about one’s level of fear of COVID-19 and the consequences of such fear. Pakpour and Griffiths^16^ opined that knowledge about the fear of COVID-19 could contribute to developing targeted education that could assist people in overcoming and/or using the fear of COVID-19 to promote preventive health behaviors. There is also a need to provide psychotherapy for those who have suffered from the negative effects of COVID-19.

Our hypothesis of serial mediation was also supported. We observed positive associations between the fear of COVID-19 and post-traumatic stress symptoms as well as psychological distress in this study, implying that participants with high levels of the fear of COVID-19 were more likely to report increased levels of post-traumatic stress symptoms and psychological distress. The results show that it is possible to experience post-traumatic stress symptoms and psychological distress concurrently, and both in combination could serially mediate (in any order that is) the relationship between the fear of COVID-19 and preventive health behavior. This is not surprising as the fear of COVID-19 has been reported to facilitate the development of a wide range of psychopathologies among those without prior mental illness.^14^ The fear of COVID-19 has also been linked to heightened psychological distress and various mental problems.^11^^,^^13^^,^^15^ Moreover, psychological distress, and post-traumatic stress symptoms caused by the COVID-19 pandemic could lead to the fear of COVID-19, forming a cyclic relationship relationship.^2^^,^^11^^,^^16^ Therapists and other mental health practitioners should pay attention to underlying psychopathologies that may be present in individuals when giving mental health care in these times of the pandemic. This is because not only are they (those with underlying mental health challenges) prone to comorbidity but are also more susceptible to disregarding preventive health behaviors.

Our hypothesis that gender will moderate the effect of the fear of COVID-19 on preventive health behavior among Nigerians was supported. Gender has a small significant moderation effect on the association of the fear of COVID-19 with preventive health behavior, suggesting that the pathway in which the fear of COVID-19 is translated into preventive health behavior is slightly higher in men than in women. This implies that the fear of COVID-19 has a slightly more positive impact on preventive health behavior when gender is male. Our results contradicted previous studies that found that women tend to report higher preventive health behavior than men because of the fear of COVID-19.^9^^,^^10^^,^^34^^,^^35^ What this means is that the influence of the fear of COVID-19 on preventive health behavior holds slightly more for males than females. Therefore, for males, the fear of COVID-19 is more likely to translate into slightly higher engagement in preventive health behavior.

In lay terms, the fear of COVID-19 could encourage preventive health behavior, slightly more among males than females. Also, the fear of COVID-19 could lead to the concurrent or separate expression of post-traumatic stress symptoms and psychological distress whereby the resultant post-traumatic stress symptoms could trigger engagement in frequent preventive health behavior, whereas psychological distress could elicit less preventive health behavior. Policies concerning the general public in times of the pandemic should reflect gender sensitivity, bear in mind the possibilities of underlying comorbidities in individuals, and reflect these as well. Health authorities should incorporate psycho-educative interventions in their response to the COVID-19 pandemic. Direct acts to reduce COVID-19 induced psychological distress and campaigns that promote adequate knowledge and attitude toward preventive health behavior are very essential.

The present study had some limitations. It used a cross-sectional design that will not allow us to draw inferences about the causal associations among variables nor provide strong evidence for causality. A longitudinal study and/or diary method would be better at demonstrating whether the correlations between the variables in this study are stable over time. Pierce and collegues^36^ warned against the use of a non-probability sampling such as snowballing technique in mental health surveys during the COVID-19 pandemic, as it could introduce bias that cannot be fully controlled. Because of the recruitment of family and friends by our participants, the survey could suffer from a possible self-selection bias, caution is suggested in interpreting the results. The sampling procedure could also account for the likely correlation between the observations due to the likelihood that participants might recruit other people that they know who were more likely to have similar perceptions of COVID-19 and engage in similar protective health behavior. Interpretation of the findings is also limited by the sample characteristics as participants were primarily young adults and individuals with access to the Internet and/smartphones, representing only a minor fraction of the general population, which could further be due to the selection bias. Future studies need to replicate the findings in other samples such as older adults or individuals without an Internet-enabled smartphone that is randomly selected, to balance any biases due to overrepresented tech-savvy younger people. Similarly, data on the response rate are unavailable thus making it difficult to assess selection bias that could also reduce the generalizability of the findings. Given the noted sampling biases, the findings must be interpreted with caution. Notwithstanding the above limitations, this study provides invaluable information on the effects of mental health status on preventive health behavior among Nigerians. The findings of this study could be used as a baseline for subsequent intervention on promoting preventive health behavior.

## CONCLUSION

In summary, this study provides some of the first empirical data examining the degree to which post-traumatic stress symptoms and psychological distress may mediate relationships between the fear of COVID-19 and preventive health behavior in a gender-sensitive manner. Our *a priori* hypotheses were supported. The relationship between the fear of COVID-19 and preventive health behavior was mediated positively by post-traumatic stress symptoms and negatively by psychological distress. Also, the fear of COVID-19 tends to slightly promote preventive health behavior among males than females. Although sampling bias means our results should be interpreted with caution, confirmation of these findings could assist in developing targeted interventions to improve preventive health behavior.

## References

[1] John Hopkins University , 2020. *Coronavirus COVID-19 Global Cases by the Center for Systems Science and Engineering (CSSE).* Available at: https://coronavirus.jhu.edu/map.html. Accessed August 9, 2020.

[2] Ahorsu DK Lin CY Imani V Saffari M Griffiths MD Pakpour AH, 2020a. The fear of COVID-19 scale: development and initial validation. Int J Ment Health Addict 1–9. doi: 10.1007/s11469-020-00270-8. PMC7100496 32226353

[3] Kimani J , 2020. The effects of COVID-19 on the health and socio-economic security of sex workers in Nairobi, Kenya: emerging intersections with HIV. Glob Public Health 15: 1073–1082. 32459578 10.1080/17441692.2020.1770831

[4] Chukwuorji JC Iorfa SK, 2020. Commentary on the coronavirus pandemic: Nigeria. Psychol Trauma Theory Res Pract Policy 12: S188–S190. doi: 10.1037/tra0000786. 32551760

[5] Olatunji OS Ayandele O Ashirudeen D Olaniru OS, 2020. ‘Infodemic’ in a pandemic: COVID-19 conspiracy theories in an African country. Social Health and Behavior 3: 152–157.

[6] World Health Organization (WHO), 2020. *Coronavirus Disease (COVID-19) Advice for the Public.* Available at: https://www.who.int/emergencies/diseases/novel-coronavirus-2019/advice-for-public. Accessed June 12, 2020.

[7] Ogoina D, 2020. COVID-19: the need for rational use of face masks in Nigeria. Am J Trop Med Hyg 103: 33–34. 32419693 10.4269/ajtmh.20-0433 PMC7356454

[8] Zhang F Shang Z Ma H Jia Y Sun L Guo X Liu N, 2020. High risk of infection caused posttraumatic stress symptoms in individuals with poor sleep quality: a study on influence of coronavirus disease (COVID-19) in China. MedRxiv. doi: 10.1101/2020.03.22.20034504.

[9] Iorfa SK , 2020. COVID-19 knowledge, risk perception and precautionary behaviour among Nigerians: a moderated mediation approach. Front Psychol 11: 566773. 33329202 10.3389/fpsyg.2020.566773 PMC7714760

[10] Olaimat AN Aolymat I Elsahoryi N Shahbaz HM Holley RA, 2020. Attitudes, anxiety, and behavioral practices regarding COVID-19 among university students in Jordan: a cross-sectional study. Am J Trop Med Hyg 103: 1177–1183. 32662398 10.4269/ajtmh.20-0418 PMC7470553

[11] Satici B Gocet-Tekin E Deniz ME Satici SA, 2020. Adaptation of the fear of COVID-19 scale: its association with psychological distress and life satisfaction in Turkey. Int J Ment Health Addict. doi: 10.1007/s11469-020-00294-0. PMC7207987 32395095

[12] Taylor S, 2019. The Psychology of Pandemics: Preparing for the Next Global Outbreak of Infectious Disease. Newcastle upon Tyne, UK: Cambridge Scholars Publishing.

[13] Jahanshahi AA Dinani MM Madavani AN Li J Zhang SX, 2020. The distress of Iranian adults during the COVID-19 pandemic-more distressed than the Chinese and with different predictors. MedRxiv 1–6. doi: 10.1101/2020.04.03.20052571. PMC7189859 32360603

[14] Shigemura J Ursano RJ Morganstein JC Kurosawa M Benedek DM, 2020. Public responses to the novel 2019 coronavirus (2019-nCoV) in Japan: mental health consequences and target populations. Psychiatry Clin Neurosci 74: e281. 10.1111/pcn.12988 PMC7168047 32034840

[15] Harper CA Satchell LP Fido D Latzman RD, 2020. Functional fear predicts public health compliance in the COVID-19 pandemic. Int J Ment Health Addict 1–14. doi: 10.1007/s11469-020-00281-5. PMC7185265 32346359

[16] Pakpour AH Griffiths MD, 2020. The fear of COVID-19 and its role in preventive behaviors. Journal of Concurrent Disorders 2: 58–63.

[17] De Coninck D d’Haenens L Matthijs K, 2020. Perceived vulnerability to disease and attitudes towards public health measures: COVID-19 in Flanders, Belgium. Pers Individ Dif 166: 110220. 32834279 10.1016/j.paid.2020.110220 PMC7327450

[18] Kuper-Smith B Doppelhofer L Oganian Y Rosenblau G Korn C, 2020. Optimistic beliefs about the personal impact of COVID-19. PsyArXiv. doi: 10.31234/osf.io/epcyb.

[19] Goldberg DP Gater R Sartorius N Ustun TB Piccinelli M Gureje O Rutter C, 1997. The validity of two versions of the GHQ in the WHO study of mental illness in general health care. Psychol Med 27: 191–197. 9122299 10.1017/s0033291796004242

[20] Mohammed A , 2015. An evaluation of psychological distress and social support of survivors and contacts of ebola virus disease infection and their relatives in Lagos, Nigeria: a cross sectional study – 2014. BMC Public Health 15: 824. 26307047 10.1186/s12889-015-2167-6 PMC4550041

[21] Jalloh MF , 2018. Impact of Ebola experiences and risk perceptions on mental health in Sierra Leone, July 2015. BMJ Glob Health 3: e000471. 10.1136/bmjgh-2017-000471 PMC5873549 29607096

[22] American Psychiatric Association , 2013. Diagnostic and Statistical Manual of Mental Disorders, 5th Edition (DSM-5). Washington, DC: American Psychiatric Association.

[23] Fekih-Romdhane F Ghrissi F Abbassi B Cherif W Cheour M, 2020. Prevalence and predictors of PTSD during the COVID-19 pandemic: findings from a Tunisian community sample. Psychiatry Res 290: 113131. 32485488 10.1016/j.psychres.2020.113131 PMC7255192

[24] Henrich J Heine SJ Norenzayan A, 2010. Most people are not WEIRD. Nature 466: 29. 20595995 10.1038/466029a

[25] Ayandele O , 2020. The Infectious Diseases Preventive Health Behavior Scale (ID-PHBS): development and validation with an African sample. MedRxiv. SSRN, 3776458. doi: 10.2139/ssrn.3776458.

[26] Gureje O Obikoya B, 1990. The GHQ-12 as a screening tool in a primary health setting. Soc Psychiatry Psychiatr Epidemiol 25: 276–280. 2237610 10.1007/BF00788650

[27] Thoresen S Tambs K Hussain A Heir T Johansen VA Bisson JI, 2010. Brief measure of posttraumatic stress reactions: impact of Event Scale-6. Soc Psychiat Epidemiol 45: 405–412. 10.1007/s00127-009-0073-x 19479171

[28] Belsley DA, 1982. Assessing the presence of harmful collinearity and others forms of weak data through a test for signal-to-noise. J Econom 20: 211–253.

[29] Brown TA, 2015. Confirmatory Factor Analysis for Applied Research (2 ed.). New York, NY: The Guilford.

[30] Hu L Bentler P, 1999. Cutoff criteria for fit indexes in covariance structure analysis: conventional criteria versus new alternatives. Struct Equ Modeling 1: 1–55.

[31] Gallucci M, 2019. *jAMM: jamovi Advanced Mediation Models [jamovi module].* Available at: https://jamovi-amm.github.io. Accessed November 15, 2020.

[32] Lazarus RS, 1991. Progress on a cognitive-motivational-relational theory of emotion. Am Psychol 46: 819–834. 1928936 10.1037//0003-066x.46.8.819

[33] Yang JZ Chu H, 2018. Who is afraid of the Ebola outbreak? The influence of discrete emotions on risk perception. J Risk Res 21: 834–853. doi: 10.1080/13669877.2016.124 7378.

[34] Ahorsu DK , 2020b. Associations between fear of COVID-19, mental health, and preventive behaviours across pregnant women and husbands: an actor-partner interdependence modelling. Int J Ment Health Addict 1–15. doi: 10.1007/s11469-020-00340-x. PMC7289236 32837427

[35] Olapegba PO , 2020. COVID-19 knowledge and perceptions in Nigeria. PsyArXiv. doi: 10.31234/osf.io/j356x.

[36] Pierce M , 2020. Says who? The significance of sampling in mental health surveys during COVID-19. Lancet Psychiatry 7: 567–568. 32502467 10.1016/S2215-0366(20)30237-6 PMC7266586

